# Ophiobolin A from *Bipolaris oryzae* Perturbs Motility and Membrane Integrities of Porcine Sperm and Induces Cell Death on Mammalian Somatic Cell Lines

**DOI:** 10.3390/toxins6092857

**Published:** 2014-09-23

**Authors:** Ottó Bencsik, Tamás Papp, Máté Berta, Annamária Zana, Péter Forgó, György Dombi, Maria A. Andersson, Mirja Salkinoja-Salonen, Csaba Vágvölgyi, András Szekeres

**Affiliations:** 1 Department of Microbiology, Faculty of Science and Informatics, University of Szeged, Közép fasor 52, Szeged H-6726, Hungary; E-Mails: bencsikotto@gmail.com (O.B.); pappt@bio.u-szeged.hu (T.P.); bertamate91@gmail.com (M.B.); mucor1959@gmail.com (C.V.); 2 Institute of Pharmaceutical Analysis, University of Szeged, Somogyi u. 4, Szeged H-6720, Hungary; E-Mails: zanaa@pharm.u-szeged.hu (A.Z.); forgop@ektf.hu (P.F.); g.dombi@pharm.u-szeged.hu (G.D.); 3 Department of Food and Environmental Sciences, Viikinkaari 9, University of Helsinki, Agnes Sjöbergin katu 2, Helsinki FI-00014, Finland; E-Mails: mariande@mappi.helsinki.fi (M.A.A.); mirja.salkinoja-salonen@helsinki.fi (M.S.-S.)

**Keywords:** ophiobolin A, boar sperm assay, *Bipolaris oryzae*, cell toxicity, mitochondrial dissipation

## Abstract

*Bipolaris oryzae* is a phytopathogenic fungus causing a brown spot disease in rice, and produces substance that strongly perturbs motility and membrane integrities of boar spermatozoa. The substance was isolated from the liquid culture of the fungal strain using extraction and a multi-step semi-preparative HPLC procedures. Based on the results of mass spectrometric and 2D NMR techniques, the bioactive molecule was identified as ophiobolin A, a previously described sesterterpene-type compound. The purified ophiobolin A exhibited strong motility inhibition and viability reduction on boar spermatozoa. Furthermore, it damaged the sperm mitochondria significantly at sublethal concentration by the dissipation of transmembrane potential in the mitochondrial inner membrane, while the plasma membrane permeability barrier remained intact. The study demonstrated that the cytotoxicity of ophiobolin A toward somatic cell lines is higher by 1–2 orders of magnitude compared to other mitochondriotoxic mycotoxins, and towards sperm cells unique by replacing the progressive motility by shivering tail beating at low exposure concentration.

## 1. Introduction

Filamentous fungi can produce large number of secondary metabolites with promising biological effects [1]. Molecular identification of these compounds and characterization of their biological activities are among the most important goals of studies working on the interface of chemistry and biology research [2]. The sperm motility inhibition test has been found to be an excellent tool to screen for bioactive microbial compounds and particularly for the mitochondriotoxic substances [3]. This test system was already used to investigate the adverse effects of several fungal metabolites. Peptaibol compounds, such as alamethicin and trilongin produced by *Trichoderma* strains and acrebol secreted by an *Acremonium* *exuviarum* strain, are the members of the peptaibols and showed remarkable motility inhibition on boar spermatozoa [4,5]. Enniatins and beauvericin mycotoxins produced by *Fusarium* strains also had significant effects on spermatozoa including motility inhibition and dissipation of the mitochondrial membrane potential [6]. Furthermore, the toxicity testing of *Bacillus*-containing food additives is based on sperm motility inhibition in most of the European registration dossiers, indicating the usefulness of this test for screening purposes [7]. 

Our recent research activities focused to the screening for bioactive fungal secondary metabolites possessing motility inhibition in this sperm test system. *Bipolaris oryzae* (Breda de Haan) Shoemaker is a phytopathogenic fungus causing brown spot disease in rice [8], and a rich source of several bioactive substances [9]. This strain also produces substance, which was able to inhibit the motility of boar sperms in our assay even in crude extracts of the ferment broth. Therefore, the substance has been purified with chromatographic techniques and identified as a member of ophiobolins (ophiobolin A, OPA) based on mass-spectrometry and NMR examinations.

**Figure 1:**
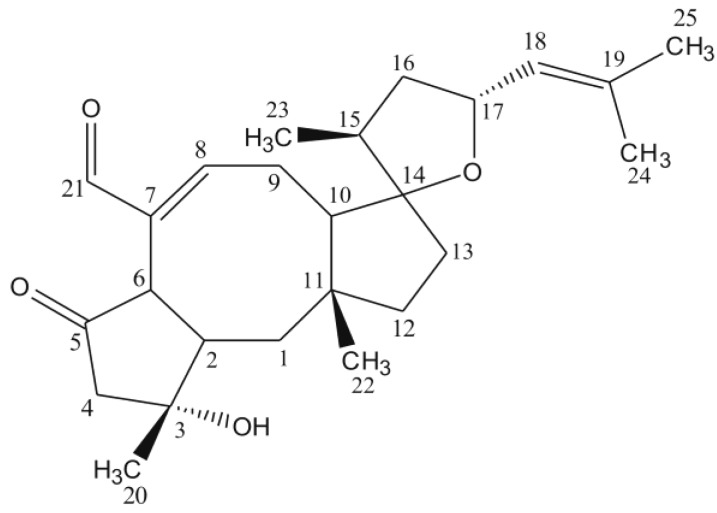
Structure of ophiobolin A.

Ophiobolins are the sesterterpene-type (C25) compounds produced by fungal species belonging to the genera *Bipolaris*, *Cochliobolus*, *Drechslera*, *Cephalosporium* and *Aspergillus* [9]. Their structure is characterized by a specific tri- or tetracyclic ring system. More than 30 of these secondary metabolites have been described to date and assigned into several subgroups [9,10,11,12,13]. These compounds show broad spectrum of biological activities, such as antimicrobial, nematocidic, cytotoxic or HIV-1 integrase inhibitory effects [9,14]. The most extensively studied representative of ophiobolins is OPA (Figure 1), the calmodulin inhibitory effect of which has been comprehensively characterized [9,15,16]. Significant reduction in the amount of different viable cells was observed after OPA treatment in ovarian cancer cell line (OVCAR3), human umbilical vein endothelial cells (HUVECs) [17] and induction of apoptotic cell death was also examined in L1210 cell line [18]. The influence of OPA on the motility and viability of mouse, rat and human sperm was reported regarding their capacitation inhibitory effects through the calmodulin antagonism [19,20,21]. Furthermore, the latest results showed that OPA is able to induce paraptosis-like cell death in human gliobastoma multiforme cells (GBM) by decreasing the big/large conductance Ca^2+^-activated K^+^ channel activity [22].

We describe here the mitochondrial toxicity and motility inhibition as well as viability reduction effect of OPA, purified and identified during our screening activities to isolate mitochondrial membrane potential (ΔΨ_m_) modifiers from microbial origin, on boar spermatozoa. Its effects on murine neuroblastoma (MNA), feline fetus lung (FFL) and porcine kidney (PK-15) cells were also tested.

## 2. Results and Discussion

**Figure 2:**
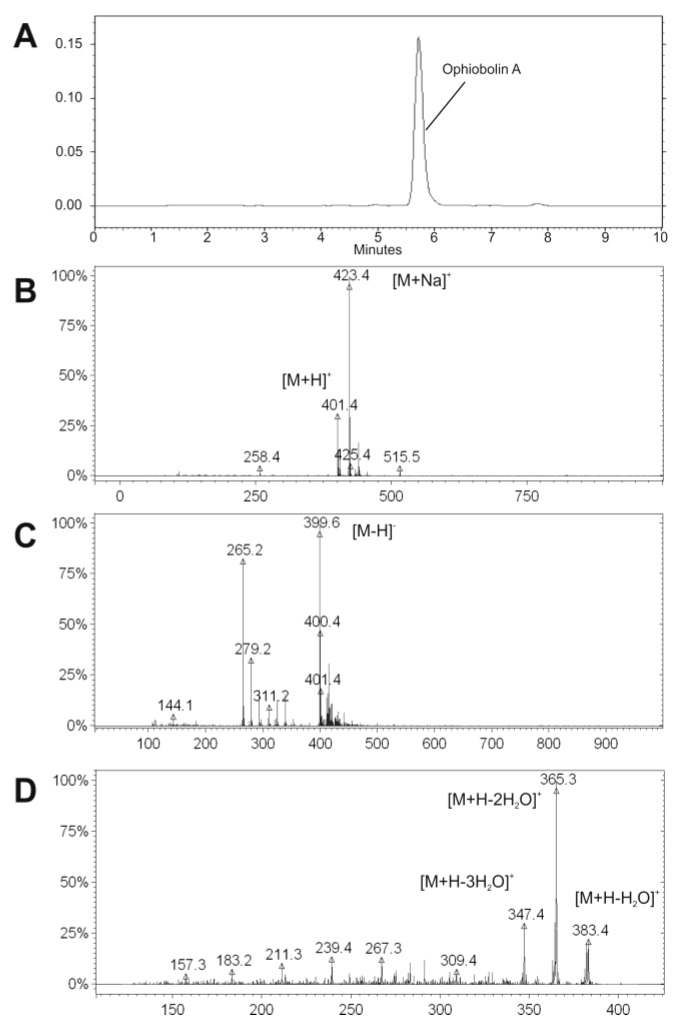
HPLC-UV chromatogram of the purified OPA at 230 nm (**A**); and the acquired mass spectra by ESI-MS in positive (**B**) and in negative mode (**C**); as well as by ESI-MS/MS after the fragmentation of *m*/*z* 401.4 in positive mode (**D**). The characteristic masses were marked.

Based on our initial screening for mitochondrial toxins, the extracted ferment broth of *Bipolaris oryzae* SZMC 13003 proved to be active using the boar sperm test system reported by Hoornstra *et al.* [3]. After the initial examinations, the toxic compound was purified and isolated as white crystals using bioactivity-guided approach. The purification was based on a multi-step semi-preparative HPLC procedure after the liquid-liquid extraction of the ferment broth. The compound was identified initially on the basis of mass spectra of electrospray ionization-mass spectrometry (ESI-MS) and fragmentation pattern of ESI-MS/MS measurements as well as on the retention time in the analytical HPLC separation compared to the further purchased reference compound (Figure 2). Furthermore, its identity was confirmed by ^1^H and ^13^C measurements as well as by detailed interpretation of a combination of ^1^H, ^1^H-COSY and HMBC spectroscopic data, which allowed the determination of the complete conformation (Figure 1) [11].

The unique properties of boar spermatozoa can be exploited for monitoring mitochondrial toxins as model targets, because these types of biological objects are insensitive to substances affecting the synthesis or regulation of proteins and nucleic acids in the cytoplasm, and all signaling systems known to operate in somatic cells have been found also in spermatozoa [3].

Furthermore, their physiological characters including their motility are influenced mainly by membrane potentials and ion fluxes. Thus, the change of sperm motility is able to sensitively indicate the substances causing mitochondrial membrane depolarization even at sublethal doses, which is undetectable with other cell lines [3]. Motility of the spermatozoa was judged after exposure of 30 min and 24 h at room temperature with minor modification of a previously described method [23,24]. OPA inhibited sperm motility after one day of exposure at 1/10 of the concentration giving inhibition visible already after 30 min, because ≤2.5 μg mL^−1^ and 250 ng mL^−1^ concentration values were determined as endpoints (EC_50_) for short and long term exposure, respectively (Table 1).

**Figure 3:**
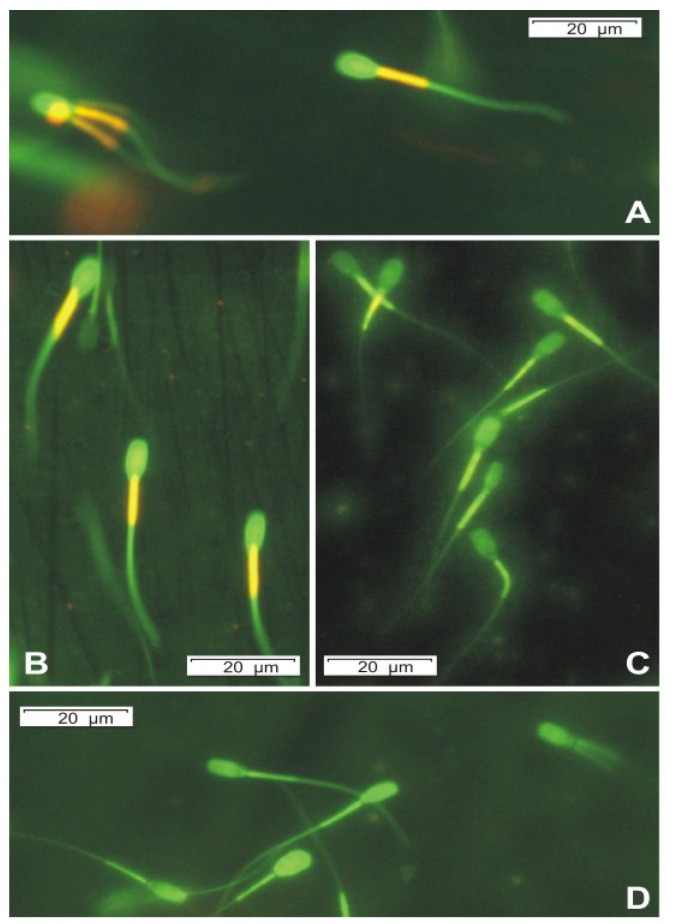
Epifluorescence micrographs of boar sperm cells stained with JC-1 at 400× magnification, the dye changes emission wavelength in response to the decreasing membrane potential by a shift from orange to green color. Top panel shows the blank exposed (reagent only) sperm cells; the mitochondrial sheath in the blank exposed spermatozoan midpiece fluorescing bright orange (**A**); Exposing the sperm cells to 125 ng mL^−1^ of OPA caused minimal difference compared to the untreated control (**B**); but the cells treated with 500 ng mL^−1^ OPA have lost *ca.* 50% of their bright fluorescing mitochondria (**C**); All mitochondria of the spermatozoan midpiece are completely depolarized after exposure to 1.25 μg mL^−1^ OPA and the sperm cells fluoresced in green only (**D**).

The membrane-permeable 5,5',6,6'-tetrachloro-1,1',3,3'-tetraethylbenzimidazolylcarbocyanine iodide (JC-1) dye is widely used in apoptosis studies to monitor mitochondrial health. It exhibits potential-dependent accumulation in mitochondria, indicated by a fluorescence emission shift from green (529 nm) to red (590 nm) [25]. Therefore, the loss of ΔΨ_m_ was seen as decrease in orange fluorescence [3]. In our cases, the staining for 2 ± 2 min with JC-1 at 37 °C was enough to get the energized mitochondria stained orange and the plasma membrane green. This short staining was empirically optimized for getting the most reproducible differences between vehicle and toxin exposed sperm cells in Hoornstra *et al.* [3]. After 2 min incubation, OPA completely depolarized the mitochondrial membrane of the sperm cells at the concentration of 1.25 μg mL^−1^ and the exposed cells have already lost approximately 50% of their bright fluorescing mitochondria at the concentration of 500 ng mL^−1^ (Figure 3, Table 1).

**Table 1:** Comparison of toxic endpoints of OPA and different mycotoxins on boar spermatozoa and on a porcine kidney cell line PK-15.

Compound name	Toxic endpoint EC_50_ μg mL^−1^ of test cell suspension							Ref.
	Boar spermatozoa						PK-15 cells	
	Motility inhibition (30 min)	Motility inhibition (24 h)	Shivering tail beating induction	Dissipation of ΔΨ_m_	Permeability to PI on static cells	Permeability to PI on motile cells	Cytotoxicity	
OPA	2.5	0.25	0.5	0.5	1.6	1.6	0.1	
Alamethicin	1	0.2	None	0.2	0.2	≤0.5	8	[4]
TrilonginBI-BIV	nd.	0.5	None	0.5	0.5	nd.	5	[4]
Acrebol	2	0.1	None	0.8	>4	nd.	≥10	[5]
Enniatin B	>10	5	None	5	>50	>20	60	[6]

nd.—no data available.

**Figure 4:**
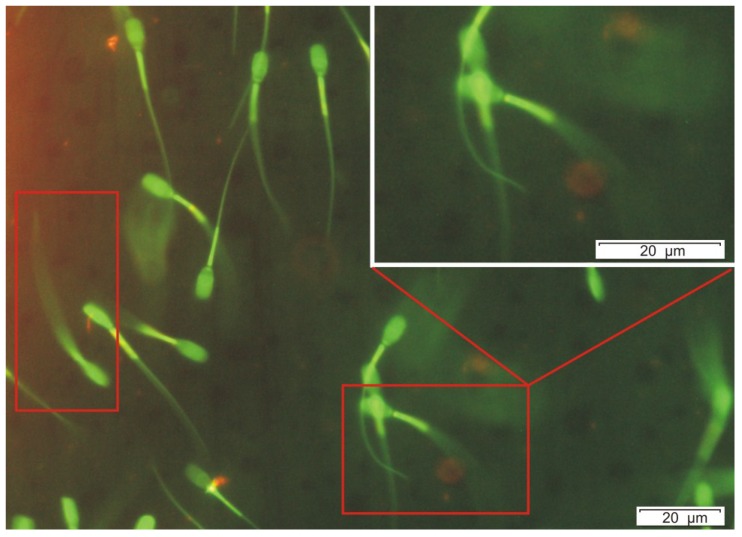
Special effect of OPA (500 ng mL^−1^, 400× magnification) on the exposed sperm cells, which showed an accelerated shivering tail beating with low amplitude, the angle between the tail beats is below 10°. The shivering tail is visible as a continuous green area and resulted in no progressive motility during the fluorescence microscopic examinations (marked with red rectangles). Microscopic area containing shivering sperm cell is enlarged at top right of the picture.

Furthermore, as a special effect, the exposed sperm cells showed in several cases an unexpected accelerated shivering mode of tail motion during the fluorescence microscopic examinations and their hyperactivity was observed (Figure 4). This motility change seems to be comparable to the phenomenon showed during the normal fertilization process, where sperm tails must switch from an easy, symmetrical beating to a frenetic whip like lashing to penetrate an egg’s protective coating [19]. The shivering tail beating exhibited by these cells with depolarized mitochondria show that the plasma membrane is intact and the cellular energy metabolism provides enough energy to motorize the sperm tail to beat. This phenomenon may indicate that the cell still produce ATP and NADH in the cytoplasm after the OPA treatment, which differs from the effect caused by cation channel forming alamethicin, which depletes the sperm cells from motility, plasma membrane integrity as well as NADH and ATP production at similar concentrations [5]. To confirm the effect of OPA on the plasma membrane permeability, the viability staining with propidium iodide (PI) and calcein-AM was performed after one day incubation at room temperature, (similar conditions as the JC-1 staining) where the EC_50_ value appeared at 1.6 μg mL^−1^, over twice the concentrations that depolarized the mitochondria (Table 1).

To test the effect of OPA exposure on sperm cells in physiological conditions (swimming at 37 °C) the plasma membrane disruption on motile sperm cells was measured with the viability PI stain, where the loss of plasma membrane integrity was detected as appearance of red color stained nuclei in the damaged cells. In this assay, sperm cells were exposed to OPA at 37 °C and motility of the exposed sperm cells was induced and maintained for 2 h [26]. The EC_50_ value fell in the range of 800 ng mL^−1^ to 1600 ng mL^−1^ (viability: 38%–73%, Table 1) and over this range the loss of the permeability barrier to PI was determined at all of the examined concentration level [27]. This result indicated that exposure to OPA in physiological conditions (as motile at 37 °C) already after 2 h (disrupting the plasma membrane) was lethal to the sperm cells in concentrations below 2000 ng/mL.

Similarly to the effect seen in boar spermatozoa, OPA inhibited the motility and reduced the viability of mouse sperms in the work of Zeng *et al.*, and, besides these effects, the inhibition of sperm capacitation was also observed [19]. OPA has been also reported to cause alteration of membrane permeability in plants including the modification of ion leakage [28] and the inhibition of proton extrusion in cells, which was the result of an effect on the permeability of the plasma membrane to potassium [29]. Bury *et al.* [22] observed increases in intracellular calcium concentration in OPA-treated GMB cells, because this toxin blocks the BKCa channels present on the cell membrane, mitochondria and ER, which induces an increase in the intracellular K^+^ concentration causing a progressive increase in the Ca^2+^ through any compensatory mechanisms to maintain homeostasis. In our case, we hypothesized that the higher concentrations of OPA used in the mitochondrial membrane depolarization assay seem to influence also the intracellular Ca^2+^ level through the ΔΨ_m_ dissipation as in GMB cells, but with lower efficiency due to the shorter incubation period, which probably served as a general hyperactivation effect in several sperms besides the already described special calmodulin inhibition. 

The toxic effects on porcine sperm cells of purified OPA were compared to four known toxins affecting different subcellular target sites (Table 1). The compounds in Table 1 produced also by filamentous fungi containing the mitochondria targeting toxins enniatin B [30], acrebol [5], the cation channel forming alamethicin [31] and trilongins [4]. Staining the sperm cells with the membrane potential sensitive dye JC-1, the live-dead stain calcein-AM and the death indicator PI divides the sperm motility inhibiting toxins in two groups. OPA belongs to the group that depolarizes the mitochondria, while the plasma membrane remained intact (acrebol, enniatin B, OPA). It is different from the group of toxins represented by the channel forming molecules (alamethicin, trilongins) that depolarize the mitochondria and disrupt the plasma membrane permeability barrier at similar concentrations (Table 1). Furthermore, OPA toxicity pattern show remarkable differences from the reported ion carrier toxins (e.g., cereulide, valinomycin) and from acrebol and enniatin B by not inducing visible hyperpolarisation of the sperm head [3,5]. Summarizing the results of the sperm cell assays, it could be concluded that the effects of OPA showed a continuous rather than sharp stepwise character: it inhibited the motility at a very low concentration; dissipated the mitochondrial membrane potential at mid-level concentration; and depleted plasma membrane integrity at moderately higher concentration. However, these values fall into a narrow interval ranging from 250 ng mL^−1^ to 1600 ng mL^−1^. Moreover, it is important to consider that OPA exerts a significantly rapid action of sperm cells exposed in physiological conditions (swimming at 37 °C), because the lethal damage indicated by loss of plasma membrane integrity was induced at low concentration (<1 μg mL^−1^) within a short period allowing to distinguish it from other sperm toxic substances. This unique pattern of action may be useful tool when investigating biological activities of other ophiobolins and structurally similar toxins. Furthermore, exposure at room temperature to a low concentration (<1 μg mL^−1^) of OPA induced in sperm cells a shivering tail beating with depolarisation of mitochondria (Figure 4). Thus OPA appears to affect differently on the two kinds of sperm flagellar motility, the shivering and the progressive motility [32]. Therefore, OPA may be used as reference to distinguish toxins blocking the progressive sperm motility, but not the shivering beating.

**Figure 5:**
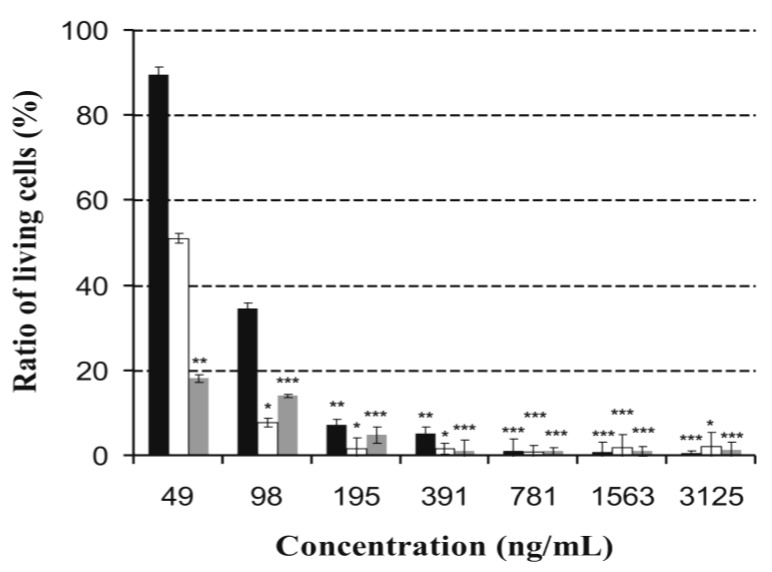
Toxic effect of ophiobolin A in different concentrations on feline fetus lung- (white bars), murine neuroblastoma- (grey bars), and porcine kidney somatic cell lines (black bars). Significance levels of the differences from the control are marked with asterisks: ***, *p* < 0.001, **, *p* < 0.01, *, *p* < 0.05.

Viability assays, based on resazurin-resorufin transformation, represent excellent correlation to generally used reference assays, such as formazan- and tritiated thymidine based methods. In our experiments, proliferating murine neuroblastoma (MNA) [33], feline fetus lung (FFL) [5] and porcine kidney (PK-15) [34] cells were treated with OPA. It proved to be highly toxic to cell proliferation in the examined cell lines and showed concentration-dependent kinetics (Figure 5). The IC_50_ values were different on the tested cell lines, less than 48.8 ng mL^−1^ on MNA, 48.8 ng mL^−1^ on FFL and between 48.8 ng mL^−1^ and 97.6 ng mL^−1^ on PK-15. These values were remarkably less than the concentrations causing sperm toxicity and motility inhibition. Our results show that OPA inhibited both sperm motility and somatic cell proliferation indicating that OPA may have an influence on both synthesis and translation of mRNA both in cytoplasm and mitochondria and/or on other mitochondrial functions [35].

When OVCAR3 and HUVECs cells were treated with OPA a significant reduction in the amount of viable cells was also observed with IC_50_ value of 112 μg mL^−1^ and 32 μg mL^−1^, respectively, and demonstrated a lack of tumor specificity [17]. Furthermore, it induced cell death on L1210 cell line in the range of 4–400 μg mL^−1^ in a concentration-dependent manner with an IC_50_ value of 120 μg mL^−1^ [18]. Based on our results, OPA is even more effective against MNA, FFL and PK-15 cell lines than against the previously tested HUVECs, OVCAR3 and L1210 cells. To compare the toxicity of OPA on PK-15 cell line to reported data of known fungal toxins, OPA scored lowest toxicity endpoints (Table 1). Considering the effects both on sperm motility and toxicity on PK-15 cells of these toxins, the motility was inhibited generally at lower concentrations, where the PK-15 cell proliferation was not blocked, but in the case of OPA both of them were hindered at similarly low exposure concentrations (≤1 μg mL^−1^, Table 1).

## 3. Experimental Section

### 3.1. General Experimental Procedures

^1^H NMR, ^1^H-^1^H COSY and HMBC spectra were recorded in MeOD on a Bruker Avance DRX 500 spectrometer (Bruker Biospin GmbH, Karlsruhe, Germany) at 500 MHz (^1^H) and 125 MHz (^13^C) JMOD, HSQC and NOESY measurements were recorded on a Bruker Ultrashield Plus 600 spectrometer (Bruker Biospin GmbH, Karlsruhe, Germany) at 600 MHz (^1^H) and 150 MHz (^13^C) using solvent signals as references (MeOD: δH 3.31/δC 47.6). The HSQC and HMBC experiments were optimized for 145.0 and 8.0 Hz, respectively. Gradient enhanced two-dimensional data were collected and processed with standard Bruker Topspin 3.0 software (Bruker Biospin GmbH, Karlsruhe, Germany, 2012). ESI-MS data were obtained using a Varian 500-MS ion trap mass spectrometer (Agilent, Palo Alto, CA, USA) equipped with an atmospheric pressure electrospray ionization (ESI) source. Ion source parameters: spray chamber temperature, 50 °C; drying gas (N_2_) pressure and temperature, 15 psi and 300 °C, respectively; nebulizer gas (N_2_) pressure, 25 psi; needle voltage, ±5800 V (positive and negative mode); spray shield voltage, 600 V; General parameters: maximum scan times, 5.21; μscans averaged, 3; data rate, 0.19 Hz; multiplier offset, 0; ionization control parameters: target TIC, 100%; maximum ion time, 500000 ms; Full scan parameters (negative/positive ionization): capillary voltage, 76.9 V; mass range, 50 *m*/*z*–2000 *m*/*z*; RF loading, 59%; MS2 parameters (positive ionization): capillary voltage, 76.9 V; RF loading, 59%; isolation window, 3 *m*/*z*; high mass ejection factor, 100%; waveform type, resonant; excitation storage level, 121.8 *m*/*z*; excitation amplitude, 1.62 V. Semipreparative normal phase HPLC separations were performed using a K-1000 Quaternary pump (Knauer, Berlin, Germany) and a Fractomax FR5 (Pharmacia, Freiburg, Germany) fraction collector equipped with an ECO 15/450V3K glass column (YMC, Dinslaken, Germany) filled with Kieselgel 60 (Merck, Budapest, Hungary), while semipreparative reverse phase HPLC runs were carried out on a Shimadzu LC-10 (Shimadzu, Duisburg, Germany) system (controller, SCL-10AVP; quaternary pump, LC-10ADVP; UV-VIS detector, SPD-10AVP; fraction collector, FRC-10A) using a Serva ODS 10 × 450 mm with 5 μm particle size. Analytical HPLC measurements were implemented on a Shimadzu LC-20 (Shimadzu, Duisburg, Germany) system (controller, CBM-20A; two pumps LC-20AD; degasser, DGU-14A; autosampler, SIL-10ADVP; column thermostat, CTO-10ASVP, detector, SPD-10AVP) with a reversed phase Phenomenex Prodigy C_18_ (150 × 4.6 mm, 3 μm) column (Gen-Lab, Budapest, Hungary) and a Phenomenex Prodigy KJ0-4282 guard (Gen-Lab, SCL-10AVP, Budapest, Hungary).

### 3.2. Strain and Culture Conditions

*Bipolaris oryzae* strain SZMC 13003 strain was obtained from Szeged Microbiological Collection (Szeged, Hungary) (http://www2.sci.u-szeged.hu/microbiology/collection.htm). The fungal strain was pre-cultured on slants of potato dextrose agar at 25 °C for 10 days. Fermentation was carried out in Erlenmeyer flasks (VWR, Budapest, Hungary), containing a total of 2600 mL of potato dextrose broth (PDB) medium (VWR, Hungary). Spore inoculum was prepared in sterile, distilled H_2_O to give a final spore suspension of 1 × 10^6^ mL^−1^. Each flask was inoculated with 5.0 mL of the spore inoculum and incubated for 12 days at 28 °C on a rotary shaker (VWR, Budapest, Hungary) (160 rpm).

### 3.3. Extraction and Isolation

Fungal cultures were filtered sequentially using a cheese cloth filter (VWR, Budapest, Hungary) and a Millipore cellulose acetate (0.22 μm) filter (Merck, Budapest, Hungary). Then the filtrate was extracted two times with equal volumes of ethyl acetate (EtOAc) and the pooled organic phases were evaporated to dryness under vacuum to afford a crude extract (1350 mg, purity: 12.9%) which was fractionated by semipreparative silica gel column chromatography (Knauer, Berlin, Germany) using an isocratic EtOAc/n-hexane eluent mixture (5:5, *v*/*v*, 2 mL min^−1^). After the evaporation, the pooled fractions eluted in the range of 14–24 min (970 mg, purity: 14.8%) were separated by semipreparative reverse phase column chromatography eluting with 3:7 H_2_O/MeCN (3 mL min^−1^). The resulting subfractions (30 mg, purity: 70.5%) were combined and further purified on the same stationary phase with the mobile phase of 5:5 H_2_O/MeCN (3 mL min^−1^) to afford OPA (9.2 mg, purity: 95.3%, 32–35 min). During the purification procedure the purity and the content of OPA were tested by analytical HPLC measurements at a wavelength of 230 nm.

### 3.4. Sperm Motility Inhibition Assay

#### 3.4.1. Examination of Progressive and Rapid Motility

The sperm motility inhibition assay was done as reported by Andersson *et al.* [24]. Commercial boar semen obtained from Figen Oy Finland extended in MR-A (Kubus, S.A., Madrid, Spain) containing 27 × 10^6^ spermatozoa·mL^−1^ was used. The purified OPA was diluted by serial dilutions in methanol obtaining two ranges of concentrations with 4-4 levels from 2500 μg mL^−1^ to 250.0 μg mL^−1^ and from 50 μg mL^−1^ to 5.0 μg mL^−1^, for short and long time exposures, respectively. Twenty μL of each dilution was administered to 2 mL of MR-A-diluted boar semen resulting approximately 100 times dilution of the original stock solutions; 20 μL of methanol was added to spermatozoa in the same way as a blank assay. The samples were incubated for 30 min and 24 h at room temperature for short and long term exposure assays, respectively. After exposure, motility was induced in the sperm cells by incubation in 37 °C for 5 min and shaking to provide the sperm cells with oxygen. Sperm motility, *i.e.*, progressive and rapid motility was assessed in phase contrast microsope (Olympus CKX41 and Software CellSens standard version 11.0.06, Olympus Soft Imaging Solutions GmbH, Munster, Germany, 2009–2012) by estimating the proportion of spermatozoa exhibiting high amplitude in tail beating. For the human eye looking in the microscope (Olympus CKX41, Munster, Germany), progressive and rapidly motile sperm cells looks like sperm cells having two separate tails, the angle between the tails being ≥40°. The amount of sperm cells exhibiting this kind of two separate tails was calculated using the CellSense standard software. The assays were performed in triplicate.

#### 3.4.2. Calibration of the Assay

The above described subjective motility assay was calibrated with an automatic Hamilton Thorne sperm motility analyzer (HTM-S, ver. 7.2; Hamilton-Thorn Research, Danvers, MA, USA). Two hundred samples of extended boar semen exposed to motility inhibiting substances were tested in parallel both with subjective method and automatic sperm motility analyzer (Hamilton-Thorn Research, Danvers, MA, USA). From the full sample set 73 samples showed a 50% decrease in rapid and progressive motility as subjectively estimated loss of high amplitude tail beating and as a decrease in progressive and rapid motility measured by the sperm analyzer. Both methods indicated no decrease in tail beating or rapid motility for 125 samples. Two samples showed slightly decreased rapid motility measured with the sperm analyzer whereas no decline was observed as the subjective estimated tail beating. Thus the protocol for assessing sperm tail beating as a measure of motility inhibition gave 99% similarity to measurements of the proportion of rapidly motile spermatozoa done with Hamilton Thorne sperm analyzer (HTM-S, ver. 7.2; Hamilton-Thorn Research, Danvers, MA, USA) Toxicity was expressed as the endpoint concentration of the OPA solution, where the progressive and rapid motility of the sperm cells were <50% of the vehicle exposed sperm cells. The assay (24 h exposure) was calibrated with triclosan (Sigma Chemical Co., St. Louis, MO, USA) in 10 parallel tests, the EC_50_ was 1 μg mL^−1^ (SD ± 0.2).

### 3.5. Membrane Integrity Disruption Assays

Cell membrane permeability assay on motile sperm cells was inspected by staining with PI (Molecular Probes, Eugene, OR, USA) in sperm cells induced to swim by shaking at 37 °C. One hundred and fifty μL aliquots of commercial extended boar semen with a density of 27 × 10^6^ sperms mL^−1^ were pipetted into microtiter plate wells and serially diluted methanolic solution of OPA were added in the concentration range of 200 ng mL^−1^ to 4 μg mL^−1^ including 6 levels and an additional methanol blank, and pre-incubated for 2 h on orbital shaker (Innova 5000 new Brunswick Scientific, Enfield, CT, USA) (160 rpm) at 37 °C. Four parallel measurements were done for each dilution. One ml PI solution (1 mg mL^−1^ in Dulbeco’s phosphate buffered saline) was mixed with 3 mL of semen Dulbeco’s phosphate buffered saline. One hundred and fifty μL of this stain mixture was added to 150 μL of exposed boar semen. This suspension was incubated for 15 min at 37 °C in dark and measured using a microplate reader (Fluoroskan Ascent, Thermo Scientific, Vantaa, Finland) at excitation and emission wavelengths of 544 nm and 600 nm, respectively. For observing plasma membrane damage by PI staining frozen-thawed semen was used as a positive control (for mortality of 100%) representing the maximal fluorescence emitted by the cells permeable to PI and Dulbeco’s phosphate buffered saline was applied as background control (blank). Loss of viability in the sample was calculated as described in Alm *et al.* [26]. The frozen sample represented 100% loss of viability. The assay was calibrated with triclosan (Sigma Chemical Co., St. Louis, MO, USA), in 5 parallel tests, the EC_50_ was 2 μg mL^−1^ (SD ± 0.6). Cell membrane permeability assay on static sperm cells at room temperature was carried out by viability staining with PI and calcein-AM (Molecular Probes, Eugene, OR, USA) according to Hoornstra *et al.* [3] in same concentration range and levels described above.

The dissipation of the ΔΨ_m_ on static cells at room temperature was visualized by 5,5',6,6'-tetrachloro-1,1',3,3'-tetraethylbenzimidazolylcarbocyanine iodide (JC-1) (Molecular Probes, Eugene, OR, USA) staining according to Hoornstra *et al.* [3] with minor modifications as follows: 200 μL of the boar semen exposed with the final OPA concentration of 2.5 μg mL^−1^, 1.25 μg mL^−1^, 0.5 μg mL^−1^ and 0.125 μg mL^−1^ was incubated at 37 °C for 2 min, stained with 0.3 μL of JC-1 (1 mg mL^−1^ in DMSO) followed by another 2 min at 37 °C and viewed using an epifluorescence microscope (Eclipse E600, Nikon, Tokyo, Japan). All assays (PI + CAM and JC-1 staining) were performed in triplicate and for each sample five microscopic fields containing *ca.* 20 cells were inspected.

### 3.6. Mammalian Somatic Cell Toxicity Assays

Cytotoxicity against somatic cells was tested with feline fetus lung cells (FFL) cultivated as described by Andersson *et al.* [5,23] and murine neuroblastoma cells (MNA) as described by Kulonen *et al.* [33] as well as porcine kidney tubular epithelial cell line (PK-15) [34], cultured as reported earlier. All somatic cell lines were maintained in an atmosphere of 95% air, 5% CO_2_ and 95% relative humidity at 37 °C on RPMI 1640 (complete medium) in a cell culture cabinet (Heracell 150i; Thermo Fisher Scientific, Vantaa, Finland). The detached cells were seeded onto 96 well microtiter plates and exposed to the purified OPA, the final concentration of which ranged from 48.8 ng mL^−1^ to 25 μg mL^−1^ containing 10 levels in methanol. After incubating the plates for one day at 37 °C, inhibition of cell proliferation was inspected in phase contrast microscope. EC_100_ for inhibition of proliferation was easy to observe as absence of cell monolayer and absence of intact cells in the well. EC_0_ was also easy to observe as an intact cell monolayer indistinguishable from the control. In microscope the determining of EC_50_ was more difficult. To achieve a more exact value for EC_50_ the inhibition ability of resazurin reduction was investigated. Therefore, after the treatement the plates were incubated for three days at 37 °C and 10 μL resazurin (Sigma Chemical Co., St. Louis, MO, USA) (400 μg mL^−1^ in normal saline) was added into the wells and the plates were incubated again for 2 h under the same conditions. Then, the plates were analyzed by a microtiter plate reader (Fluoroskan Ascent, Thermo Scientific, Vantaa, Finland) at the excitation and emission wavelengths of 544 nm and 590 nm, respectively. The toxicity was expressed as the lowest concentrations where the ratio of the living cells was less than 50% (EC_50_). All of the mammalian somatic cell toxicity assays were repeated three times. This EC_50_ fitted between EC_100_ and EC_0_ observed in microscope, the maximal difference between the two methods was one dilution step. The assay was calibrated with triclosan (Sigma Chemical Co., St. Louis, MO, USA), the EC_50_ in 10 parallel tests was 9.4 μg mL^−1^ (SD ± 3.4).

### 3.7. Statistical Analysis

All the statistical analyses were performed using GraphPad Prism version 5.01 for Windows (GraphPad Software, San Diego, CA, USA, 2007). The significant differences between the viability of cells treated with OPA and their respective controls at different concentration levels were determined by one-way analysis of variance with Bonferroni's multiple comparison post-test. 

## 4. Conclusions

The results of this study describe the purification of OPA produced by *Bipolaris oryzae* strain SZMC 13003 based on multistep chromatographic separation. Furthermore, the biological activities of the isolated compound on porcine sperm and different mammalian somatic cell lines were also demonstrated. OPA exhibited strong motility inhibition and viability reduction on boar spermatozoa and significantly damaged the sperm mitochondria at sublethal concentration by the dissipation of transmembrane potential in the mitochondrial inner membrane. These effects exhibited a continuous character in the range of 250 ng mL^−1^ to 1600 ng mL^−1^ such as the motility of the sperm cells was inhibited at a low concentration, the mitochondrial membrane potential was dissipated at mid-level concentration, and the plasma membrane integrity was depleted at moderately higher concentration. OPA was also highly toxic to cell proliferation in MNA, FFL and PK-15 somatic cell lines under the level causing motility inhibition on boar spermatozoa. The observed toxicity depended on the applied concentration and the most toxic effect was recorded on the MNA cells.
